# Semi-domesticated dogs as a potential reservoir for zoonotic hookworms in Bangkok, Thailand

**DOI:** 10.14202/vetworld.2020.909-915

**Published:** 2020-05-16

**Authors:** Jutamas Wongwigkan, Tawin Inpankaew

**Affiliations:** 1 Center for Agricultural Biotechnology, Kasetsart University, Kamphaeng Saen Campus, Nakhon Pathom, Thailand; 2 Center of Excellence on Agricultural Biotechnology: (AG-BIO/PERDO-CHE), Bangkok, Thailand; 3 Department of Parasitology, Faculty of Veterinary Medicine, Kasetsart University, Bangkok, Thailand

**Keywords:** Bangkok, hookworm, semi-domesticated dogs, Thailand

## Abstract

**Background and Aim:**

Hookworms are parasitic nematodes that live in the small intestine of their mammalian hosts including humans, dogs, and cats. This study was conducted to determine the prevalence and perform genetic characterization of hookworms using molecular techniques and to elucidate the risk factors associated with hookworm infections among semi-domesticated dogs residing in temples in the Bangkok Metropolitan Area, Thailand.

**Materials and Methods:**

A total of 500 fecal samples were collected from semi-domesticated dogs from 91 temples in 48 districts of Bangkok. DNA was extracted and screened using internal transcribed spacer polymerase chain reaction-restriction fragment length polymorphism. In addition, samples positive for *Ancylostoma ceylanicum* were further characterized at the haplotype level based on the analysis of the mitochondrial cytochrome oxidase-1 gene (*cox1*).

**Results:**

The prevalence of hookworm infections in semi-domesticated dogs was 6.2% (31/500). Hookworm infections were detected in temple-community dogs in 12 of 48 districts (25.0%), with Bang Khen and Lak Si districts having the highest proportion of infected dogs (22.6%). Regarding molecular characterization of hookworm species, 21 positive samples (67.74%) were infected with *A*. *ceylanicum* and 10 (32.26%) with *Ancylostoma caninum*. Characterization of *cox1* in *A*. *ceylanicum* isolates revealed the presence of a mixture of human and dog isolates.

**Conclusion:**

Semi-domesticated dogs act as a potential source of hookworm infections for human and animal populations in Bangkok, Thailand.

## Introduction

Hookworms are parasitic nematodes that live in the small intestine of their mammalian hosts such as humans, dogs, and cats. Helminthiasis, which is caused by hookworms, is the most common soil-transmitted infection and neglected tropical disease [1,2]. The major clinical signs of this disease are chronic intestinal blood loss, iron deficiency anemia, and cutaneous larva migrans. The hookworm species that cause the majority of infections in humans are *Necator americanus* and *Ancylostoma duodenale*, whereas those responsible for causing the majority of infections in dog are *Ancylostoma caninum* and *Ancylostoma ceylanicum* [3]. Furthermore, *A*. *ceylanicum* is a potential zoonotic hookworm and can infect cats and dogs throughout Asia. Hookworm infections are common throughout sub-Saharan Africa, America, South China, and Southeast Asia [4,5]. Numerous cases of hookworm infections in domestic dogs and cats have been reported in several countries such as the East Coast of the United States [6], Northern Australia tropical rainforests [7], India [8], Cambodia [9], Laos [10], and Malaysia [11,12].

In Thailand, three studies have reported the prevalence of hookworm infections including a report on dogs in an animal refuge in Nakhon Nayok (prevalence = 30.6%) [13], a study on dogs in Bangkok (Thailand) temples (prevalence = 58.0%) [14], and another study on dogs in lower Northern Thailand (prevalence=21.3%) [15]. Therefore, these and the above-mentioned reports demonstrate that dogs and cats likely serve as reservoirs for hookworms causing infections in humans. In addition to being commonly found in domestic dogs and cats, hookworms comprising at least 68 species have been reported to infect wild animals [16,17], and *A*. *ceylanicum* infections have been reported in wild canines [18].

There are numerous dogs, particularly stray and semi-domesticated dogs, in Thailand whose population is continually increasing. As reported by the Bureau of Disease Control and Veterinary Services, the semi-domesticated dog population in Bangkok is approximately 140,000. Poor hygiene practices and overpopulation of these dogs will most likely increase the rates of disease transmission. These dogs serve as reservoirs for several parasites such as *Strongyloides* spp., *Trichuris* spp., and *Ancylostoma* spp., which can cause zoonotic diseases in humans. In Thailand, semi-domesticated dogs residing in temples live close to humans, which are consequently a potential reservoir for hookworms.

In the present study, we aimed to determine the prevalence and genotype of hookworms and to elucidate the risk factors associated with hookworm infections in semi-domesticated dogs residing in temples in Bangkok using molecular techniques.

## Materials and Methods

### Ethical approval

This study was approved by the Animal Ethics Committee of Kasetsart University, Bangkok (ACKU60-VET-006).

### Study area, study period and sample collection

The sampling was conducted between June to December 2015. The Bureau of Disease Control and Veterinary Services reported stray dog population of 720,000 dogs (z) in Bangkok using the statistical theory. Using the formula 
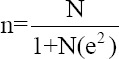
 (N = 720,000; e = 0.05), a sample size (n) of 394 was determined. However, it was decided to collect 500 samples for better representation and to account for rejected samples. The selection of temples for sample collection in Bangkok was performed in such a manner as to maximize the range of sample collection and the degree to which it represented the sample population. The districts in Bangkok differ in terms of area and population size as well as the number of temples. In other words, a single temple was sampled in a small district, whereas 2-3 temples were sampled in a bigger district, with 5-10 dogs being sampled in each temple. A fecal sample was collected from the rectum of individual dogs, transferred to a plastic bag, and maintained in a cool condition during transportation. All fecal samples were transported to the Department of Parasitology, Faculty of Veterinary Medicine, Kasetsart University, and screened for hookworms using molecular techniques. Furthermore, to record information about each dog, monks or animal caretakers were asked to complete a questionnaire related to the dog’s environment and hygiene, including the following questions: Can the dog roam freely around the temple? Does the dog receive attention from a veterinarian? and Does the dog receive vaccines or deworming program?

### DNA extraction

Genomic DNA was extracted directly from 200 mg fecal samples using a commercially available DNA extraction kit (E.Z.N.A.® Stool DNA Kit, Omega Bio-Tek Inc., Norcross, GA, USA) according to the manufacturer’s instructions. The final elution of DNA was prepared in 100 ml of elution buffer. All extracted DNA samples were stored at −20°C until polymerase chain reaction (PCR) analysis.

### Molecular characterization of hookworm species

Diagnostic PCR-restriction fragment length polymorphism (RFLP) characterization of canine hookworms was performed as described previously [9,14,19,20]. The internal transcribed spacer (ITS) regions ITS1, 5.8S, and ITS2 were used to amplify 545 bp of *Ancylostoma* genome using the primers RTGHFI (5′-CGTGCTAGTCTTCAGGACTTTG-3′) and RTABCR1 (5′-CGGGAATTGCTATAAGCAAGTGC-3′). The final reaction volume of PCR was 20 μl containing 1.25 pmol of each primer, 1 U of iTaq DNA polymerase, 10× PCR buffer, and 2 μl of DNA. The PCR conditions included heating at 94°C for 2 min, 64°C for 1 min, and 72°C for 2 min, followed by 50 cycles at 94°C for 30 s, 64°C for 30 s, 72°C for 30 s, and a final extension step at 72°C for 7 min. The amplified PCR products of RTGHF1–RTABCR1 were digested with *Rsa*l to separate *Ancylostoma tubaeforme* from *A*. *ceylanicum* and *A*. *caninum*, and *HinF*1 was used to differentiate *A*. *caninum* from *A*. *ceylanicum*. Next, 10 μl of the PCR product was digested with 5 units of a restriction endonuclease at 37°C for 6 h in a total volume of 20 ml [9,14,21]. The RFLP profiles of each sample were compared with the expected RFLP profiles for each hookworm species.

### PCR and DNA sequencing of *cox1* in *A. ceylanicum*

*A*. *ceylanicum*-positive samples according to PCR-PFLP were further characterized by amplification of the mitochondrial gene (*cox1*) at the haplotype level [9,22]. A 377 bp fragment of *cox1* in *A*. *ceylanicum* was amplified using AceyCOX1F (5′-GCTTTTGGTATTGTA-AGACAG-3′) and AceyCOX1R (5′- CTAACAACATAATAAG-TATCATG-3′). The final reaction volume of PCR of 20 μl contained 1.25 pmol of each primer, 1 U of iTaq DNA polymerase, 10× PCR buffer, and 2 μl of DNA. The cycling conditions were 95°C for 5 min, followed by 50 cycles at 94°C for 30 s, 58°C for 30 s, 72°C for 30 s, and a final extension step at 72°C for 7 min [9]. Positive samples obtained on PCR were purified using the FavorPrep™ GEL/PCR Purification Kit (Favorgen Biotech Corporation, Ping-Tung, Taiwan) and submitted for sequencing to AITbiotech (AITbiotech Pte. Ltd., Singapore).

### Phylogenetic analysis

DNA sequencing was performed using FinchTV version 1.4.0, and the results were analyzed using BLAST (National Center for Biotechnology Information). DNA sequences were aligned with *cox1* sequences using BioEdit version 7.2.5. Neighbor-joining method was conducted using Tamura-Nei parameter distance estimates, and a phylogenetic tree was constructed using Mega 6 (Pennsylvania State University, Pennsylvania, USA) (www.megasoftware.net). Bootstrap analyses were performed using 1000 replicates.

### Statistical analysis

R version 3.4.2 was used for data entry and statistical analyses. Data regarding the prevalence of hookworm infections were summarized using cross-tabulations. Univariate analysis was performed using logistic multiple regression with 95% confidence interval (CI) to determine the association between the presence of hookworm pathogens and putative risk factors such as host factors (sex and age) and location (zone).

## Results

### Prevalence of hookworm infection

**Figure-1:**
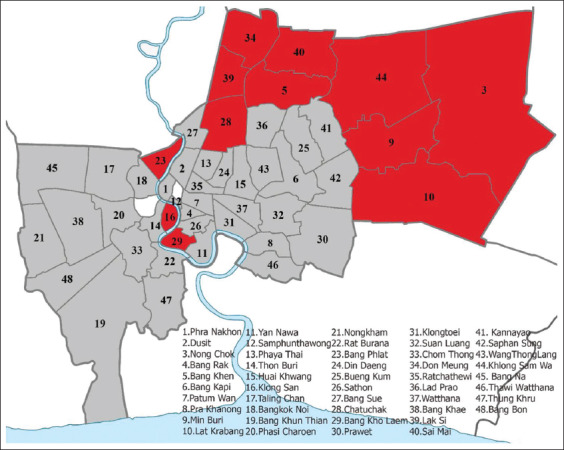
Map of hookworm-infected districts (red areas) and non-infected districts (gray areas) in Bangkok, Thailand [Source: https://upload.wikimedia.org/wikipedia/commons/4/4f/Thailand_Bangkok_location_map.png].

According to the results of the PCR-based amplification of the partial ITS gene, the prevalence of hookworm infections was only 6.2% (n = 31) among the 500 semi-domesticated dogs. Hookworm infections were detected in temple-community dogs in 12 of the 48 districts (25.0%; Figure-1), with Lak Si district having the highest proportion of hookworm-infected dogs (53.8%) followed by Klong San (33.33%) and Lat Krabang districts (27.78%; Table-1).

**Table-1:** Prevalence of hookworm spp. infections in semi-domesticated dogs residing in monasteries of Bangkok.

District	Number of dogs	Number of positive (%)
Lak Si	13	7 (53.8)
Klong San	6	2 (33.33)
LatKrabang	18	5 (27.78)
Bang KhoLaem	4	1 (25.00)
Don Mueang	13	3 (23.07)
Min Buri	7	1 (14.28)
Bang Khen	53	7 (13.20)
NongChok	8	1 (12.50)
Bang Phlat	9	1 (11.11)
Chatuchak	11	1 (9.09)
Sai Mai	20	1 (5.00)
Klong Sam Wa	21	1 (4.76)
Lad Prao	23	0 (0.00)
Saphan Sung	8	0 (0.00)
Dusit	5	0 (0.00)
Kannayao	9	0 (0.00)
BuengKum	15	0 (0.00)
Phaya Thai	10	0 (0.00)
Ratchathewi	10	0 (0.00)
Samphanthawong	4	0 (0.00)
Sathon	12	0 (0.00)
PhraKhanong	7	0 (0.00)
Bang Kapi	12	0 (0.00)
Bang Na	13	0 (0.00)
Bang sue	12	0 (0.00)
Bang Khae	13	0 (0.00)
Nongkham	12	0 (0.00)
Prawet	16	0 (0.00)
Taling Chan	9	0 (0.00)
Rat Burana	9	0 (0.00)
Khlong Toei	5	0 (0.00)
Bangkok Noi	5	0 (0.00)
HuaiKhwang	9	0 (0.00)
Bang Bon	4	0 (0.00)
Chom Thong	7	0 (0.00)
ThungKhru	16	0 (0.00)
Phasi Charoen	7	0 (0.00)
ThawiWatthana	3	0 (0.00)
Bang KhunThian	12	0 (0.00)
Din Daeng	7	0 (0.00)
Thon Buri	11	0 (0.00)
SuanLuang	2	0 (0.00)
Watthana	3	0 (0.00)
Yan Nawa	11	0 (0.00)
Wang Thong Lang	2	0 (0.00)
Bang Rak	3	0 (0.00)
PhraNakhon	7	0 (0.00)
Pathum Wan	4	0 (0.00)
Total	500	31 (6.2)

### Molecular characterization of hookworm species

According to the molecular characterization of hookworm species by RFLP, 21 of the 31 positive samples were infected with *A*. *ceylanicum* (67.7%) and 10 with *A*. *caninum* (32.3%).

### Phylogenetic analysis of *cox1* in *A. ceylanicum*

**Figure-2:**
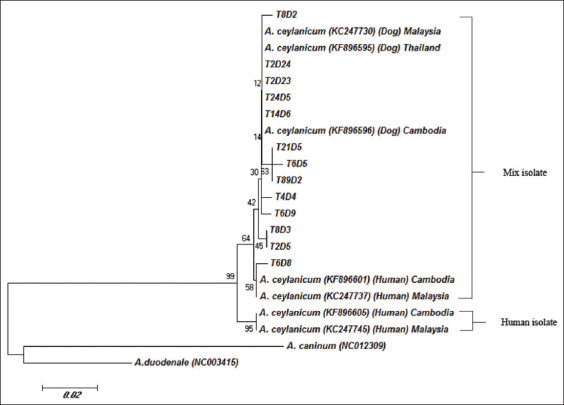
Phylogenetic analysis of hookworm based on the nucleotide sequences of a 377 bp fragment of *cox1* gene by the neighbor-joining method.

A 377 bp fragment of the cytochrome c oxidase subunit from the above-mentioned 21 samples positive for *A*. *ceylanicum* was amplified and subjected to DNA sequencing and subsequent phylogenetic analysis. The phylogenetic tree separated into three clusters with *A*. *ceylanicum* grouped together and distinct from *A*. *caninum* (GenBank accession no. NC02309) and *A*. *duodenale* (GenBank accession no. NC003415). *A*. *ceylanicum* group comprised a mixture of isolates from previously reported human isolates from Malaysia (GenBank accession nos. KC247737 and KC247745) and Cambodia (GenBank accession nos. KF896601 and KF896605) and in dog isolates from Malaysia (GenBank accession no. KC247730), Cambodia (GenBank accession no. KF896596), and Thailand (GenBank accession no. KF896595). Characterization of *cox1* in *A*. *ceylanicum* isolates revealed the presence of a mixture of human and dog isolates in all positive samples (Figure-2).

### Risk factors associated with hookworm infections in semi-domesticated dogs

Multiple logistic regression analysis of risk factors revealed an increased risk of hookworm infections in dogs living in the suburbs of Bangkok (p<0.0001, odds ratio=4.55, 95% CI=1.98-10.47). Although the prevalence of hookworm infections was the highest in dogs aged <1 year and male dogs, no significant associations were detected between the prevalence of hookworm infections and age group, sex, household, and hygiene practices (Table-2).

**Table-2:** Risk factors associated with hookworm infection.

Factors	Number of dogs	Number of positive (% positive)	p	Odds ratio	95% CI	

					Lower	Upper
Sex			0.205	0.61	0.29	1.31
Male	212	17 (8.0)				
Female	288	14 (4.8)				
Total	500	31 (6.2)				
Age			0.307	0.71	0.37	1.37
<1 year	59	7 (11.8)				
1-5 years	310	18 (5.8)				
>5 years	131	6 (4.5)				
Total	500	31 (6.2)				
Free-roaming			0.835	0.92	0.43	1.98
Yes	268	16 (5.9)				
No	232	15 (6.4)				
Total	500	31 (6.2)				
Veterinary attention			0.338	1.64	0.59	4.54
Yes	361	22 (6.0)				
No	139	9 (6.4)				
Total	500	31 (6.2)				
Rabies vaccine			0.056	0.33	0.10	1.03
Yes	344	26 (7.5)				
No	156	5 (0.03)				
Total	500	31 (6.2)				
Dewormed			0.628	1.27	0.481	3.361
Yes	123	6 (4.8)				
No	377	25 (6.6)				
Total	500	31 (6.2)				
Location			0.0001	4.55	1.98	10.47
Suburban	120	18 (15)				
Urban fringe	241	9 (3.73)				
Inner city	139	4 (2.87)				
Total	500	31 (6.2)				

## Discussion

We found a relatively low prevalence (6.2%) of hookworm infections in Thailand, which is lower than that reported previously in temple dogs in Bangkok (58.0%) [14], shelter dogs in Nakhon Nayok (30.6%) [13], and dogs in lower Northern Thailand (21.3%) [15] as well as in China (20.2%) [23,24], Laos (94.0%) [10], Malaysia (48.0-71.1%) [11,12], and Cambodia (95.7%) [9]. This finding confirmed that hookworms are pathogens that infect semi-domesticated dogs in Bangkok. The majority of semi-domesticated dogs live outdoors and roam freely, and they shed parasite eggs in public areas by which healthy animals and humans may become susceptible to infections. The previous studies have also reached a similar conclusion regarding parasites in dogs [25] and cats [26] as well as on temple grounds [27] in Bangkok. These studies have reported that 6 of 12 positive districts are in the suburban area and most of the infected districts are located adjacent to the neighboring provinces of Bangkok. These results provide important information about the presence of dog-related hookworms that may spread on temple grounds of these districts.

A case each of human hookworm infection was reported from Chachoengsao [28], Khon Kaen, and Nakhon Si Thammarat [29] as well as among Myanmar refugees living in camps along the Myanmar-Thailand border [30]. *N*. *americanus* was the most common hookworm species, with *A*. *ceylanicum* also being detected. Our findings strongly support the findings of the previous studies [9,24,31], demonstrating that the primary culprit of hookworm infections in semi-domesticated dogs was *A*. *ceylanicum*, which is one of the neglected parasitic zoonoses followed by *A*. *caninum* [32]. *A*. *ceylanicum* is an endemic and widely distributed hookworm species found in dogs and cats in Asia. Moreover, it can easily infect the intestine of human adults [33], leading to the development of eosinophilia, iron deficiency anemia, abdominal pain, and bloody diarrhea [23,34-36].

Based on molecular epidemiological data obtained from the characterization of *cox1* in *A*. *ceylanicum*, the isolates could be divided into two genetically distinct groups, with the first clade comprising isolates from humans, dogs, and cats and the second clade specific to humans [25,37]. All 21 *A*. *ceylanicum* hookworm isolates from dogs in our study grouped strongly within the zoonotic haplotype of *A*. *ceylanicum* within a subgroup comprising dog and human isolates from Malaysia, Cambodia, and Thailand; this confirmed that the transmission from dogs to humans follows the pattern established in a previous study [9].

Analysis of the risk factors for hookworm infections in humans in Thailand revealed walking barefoot and raising buffalos near the house to be definite significant risk factors [29]. Our study demonstrated that dogs living in the suburbs of Bangkok are more likely to be infected than those living in other zones (inner city and urban fringe), which is perhaps due to poor hygiene in the suburbs and nearby provinces. However, no study has confirmed this theory because in the study by Traub *et al*. [14], data were not analyzed according to location. However, we believe that this is a possible explanation for the importance of temple location as a risk factor. Further study is required to compare the distribution of hookworm infections in Bangkok. The grounds of suburban temples are generally plain, whereas city temples have cement surfaces. Because people walk barefoot and dogs defecate everywhere, the waste could more easily seep into the soil and contaminate it more than through cement ground, thereby increasing the chances of infection. These findings suggest that hygiene practices such as removal of feces and dog health care are important for the prevention and control of hookworm infections. It is necessary to conduct further studies in humans and other animals such as cats as well as obtain environmental samples to explore the relationship among humans, animals, and the environment with regard to hookworm infections.

## Conclusion

This study has provided important information regarding the status and risk of hookworm infections among semi-domesticated dogs in Bangkok. To the best of our knowledge, our study is among the first few studies to amplify *cox1*, to describe the subspecies of *A*. *ceylanicum*, which is a zoonotic isolate from semi-domesticated dogs residing in temples, and to provide evidence showing that semi-domesticated dogs act as a potential source of hookworm infections to human and animal populations in Bangkok. Therefore, it would be important to focus on the importance of zoonotic ancylostomiasis, and a “one health” approach should be adopted to control and prevent hookworm infections in Thailand.

## Authors’ Contributions

TI planned and designed the experiment and JW conducted the experiment and interpreted the results and data analysis. All authors drafted and revised the manuscript. All authors read and approved the final manuscript.
